# Learning Between the Lines: Anaesthetists’ Conceptions of the Implicit Curriculum in Postgraduate Education

**DOI:** 10.5334/pme.2224

**Published:** 2026-06-15

**Authors:** Hanna Chin, Åke Ingerman, Linda Block, Helena Odenstedt Hergès

**Affiliations:** 1 Consultant Anaesthetist and Director of Studies, Department of Anesthesiology, Surgery and Intensive Care, Sahlgrenska University Hospital, Gothenburg, Sweden; 2 Institute of Clinical Sciences, Sahlgrenska Academy, University of Gothenburg, Gothenburg, Sweden; 3 Professor, Science and Technology Education, Department of Pedagogical, Curricular and Professional Studies, University of Gothenburg, Gothenburg, Sweden; 4 Consultant Anaesthetist and Associate Professor, Department of Anesthesiology, Surgery and Intensive Care, Sahlgrenska University Hospital, Gothenburg, Sweden; 5 Consultant Anaesthetist, Associate Professor, and Vice Head (Education), Department of Anesthesiology, Surgery and Intensive Care, Sahlgrenska University Hospital, Gothenburg, Sweden

## Abstract

**Introduction:**

Competency-based and data-driven frameworks increasingly shape postgraduate anaesthesia training, yet much of professional learning occurs through unspoken cultural and relational influences—the implicit curriculum. This study examines how anaesthetists perceive and navigate these tacit aspects of learning.

**Methods:**

We conducted a phenomenographic analysis of 29 semi-structured interviews with anaesthetists from teaching hospitals in Sweden and England. Participants represented diverse training stages and educational backgrounds. Through iterative comparison and interpretation, we identified qualitatively different ways anaesthetists conceptualised learning within the social and emotional contexts of clinical work.

**Results:**

Three conceptions were identified: Orientation toward the explicit curriculum, where learning is framed by formal structures and supervision; Awareness of the implicit curriculum, where unstructured and relational learning become visible; and Engagement with the implicit curriculum, where anaesthetists actively integrate unspoken norms, values, and emotions into professional practice. Variation across five dimensions—communication, collaboration, decision-making, autonomy, and emotional intelligence—illuminated how awareness of the implicit curriculum underpins professional growth.

**Discussion:**

The implicit curriculum emerges as a lived, relational dimension of anaesthesia training rather than a hidden layer of influence. Recognising these distinct ways of understanding offers educators a framework to make tacit learning visible, foster reflective dialogue, and support the development of adaptive, self-aware professional identities across postgraduate medical training.

## Introduction

Postgraduate anaesthesia training is increasingly shaped by competency-based frameworks and data-driven technologies, promising transparency and accountability in defining progress in professional learning [1,2]. As artificial intelligence and standardisation permeate medical education, questions arise about what this emphasis makes visible—and what it may leave unseen. Within anaesthesia, where sound judgement, calm teamwork, and moral composure matter as much as clinical precision [3], much of what defines expertise may lie beyond what can be easily codified or exhaustively assessed [4].

Capabilities such as communication and collaboration, along with the management of uncertainty, are indispensable to anaesthetic practice. While aspects of these qualities can be explicitly taught, simulated, and assessed through structured approaches [5,6], their situational meaning, ethical weight, and enactment continue to develop through participation in clinical work, where expectations, values, and judgements are often tacitly modelled and negotiated [7]. The challenge, therefore, extends beyond formal incorporation into competency-based frameworks, toward understanding how these capabilities are experienced in practice. Addressing this challenge calls for conceptual frameworks that make visible the tacit and value-laden dimensions of professional learning.

To make sense of these dimensions of professional learning, medical education has drawn on a set of closely related curricular concepts, commonly described within the literature as the hidden, informal, and implicit curriculum. Although these terms often overlap in the literature [8] and in practice, distinguishing them analytically allows greater clarity. The hidden curriculum has been used to describe broader institutional and cultural forces—such as hierarchy and professional norms—that shape learning beyond formal teaching [9]. The informal curriculum refers to unscripted learning encounters that arise through day-to-day interactions in clinical settings [9]. The implicit curriculum, which is the focus of the present study, is understood here as the tacit, unspoken expectations and values that learners encounter in clinical practice, shaping what is legitimised, prioritised, or taken for granted.

The present study focuses on the implicit curriculum because it is particularly salient to understanding how anaesthetists develop professional judgement, autonomy, and relational competence in everyday clinical work. While analyses of the hidden curriculum often emphasise institutional structures, and studies of the informal curriculum highlight situated learning encounters, this focus centres on how clinicians notice, interpret, and work with tacit expectations in practice.

Previous work has highlighted how tacit influences are passed on within anaesthetic practice, including the development of non-technical skills and professional identity [10]. However, how anaesthetists themselves perceive and interpret these influences remains underexplored. This matters because what learners attend to, and how they make sense of it, shapes how they engage with their environment and develop professional capability.

To address this gap, the present study employs phenomenography to explore the qualitatively different ways anaesthetists conceive of the implicit curriculum and its role in their professional learning.

## Methods

### Study design

Phenomenography seeks to describe collective patterns of variation in how people experience and understand a phenomenon, rather than focusing on individual experiences. It is grounded in a non-dualistic epistemology, in which learning is relational—arising through interaction between learner and phenomenon—rather than a property of either alone [11].

In the present analysis, the implicit curriculum constituted the focal phenomenon. The different ways anaesthetists described and made sense of tacit norms, expectations, and values shaping postgraduate learning formed the outcome space—that is, a structured representation of how the implicit curriculum was understood within postgraduate anaesthesia training.

The interview material analysed here was generated within two phenomenographic projects examining anaesthetists’ experiences of postgraduate learning more broadly. While those studies addressed postgraduate learning in general terms, the current analysis re-engages the full dataset to explore variation in how the implicit curriculum was understood. In phenomenography, analytic significance lies in identifying recurring patterns of understanding across a collective dataset rather than in the completeness of any single individual account. Relevant understandings may therefore be expressed briefly or unevenly by participants.

When analysing experienced curriculum, both explicit and tacit dimensions are inherently present in participants’ accounts. Although implicit aspects were not foregrounded in the original analyses, the interviews nevertheless contained rich references to unspoken norms, expectations, and values. The breadth and contextual diversity of the dataset enabled variation in these understandings to be discerned despite uneven articulation at the individual level.

### Context and participants

The phenomenon under study was the implicit curriculum of postgraduate anaesthesia training, understood as a shared clinical and educational endeavour. Participants were engaged in the same disciplinary practice and oriented toward the same educational goal—becoming an anaesthetist—allowing their accounts to be treated as instances of a common phenomenon.

How the implicit curriculum was experienced and described was understood to vary according to both individual and contextual circumstances. Participants were therefore purposively recruited to represent a range of experiences of postgraduate anaesthesia training, reflecting differences among individuals (e.g. prior experience and professional role) and differences in training contexts (e.g. supervision, clinical situations, and organisational arrangements), including training in Sweden and England.

These differences were not treated as analytic categories, but as part of the experiential conditions through which the implicit curriculum was encountered. All interviews were analysed together as a single dataset. Participant characteristics are presented in Table 1, and contextual characteristics in Table 2.

**Table 1:** Participant distribution by training level and country.


TRAINING LEVEL	NUMBER OF PARTICIPANTS	ANAESTHETISTS IN SWEDEN	ANAESTHETISTS IN ENGLAND

Year 1–2	8	6	2

Year 3–7	10	4	6

Consultants	11	5	6


Table showing participant distribution by training level and country.

**Table 2:** Contextual features of postgraduate anaesthesia training in England and Sweden.


DIMENSION	ENGLAND	SWEDEN

Regulatory locus	Curriculum developed by the Royal College of Anaesthetists and approved by the General Medical Council.	National specialist training regulation issued by the National Board of Health and Welfare, with specialty-specific recommendations developed by the Swedish Society of Anaesthesia and Intensive Care.

Supervision structure	Named educational supervisor and clinical supervisors; structured appraisal and documentation processes.	Principal supervisor and director of studies; supervision embedded within clinical service and local structures.

Assessment culture	Use of workplace-based assessments alongside national summative examinations (e.g., FRCA) within a nationally defined framework.	Assessment of competence embedded in supervised clinical service, together with completion and documentation of mandatory educational activities, including required courses.

Progression logic	Formal review of progression conducted within a nationally standardised Annual Review of Competence Progression (ARCP) process.	Formal review of progression conducted locally through documented fulfilment of nationally regulated learning outcomes, typically discussed within a local specialist review meeting (ST-kollegium).


### Data collection

Semi-structured interviews were conducted to explore anaesthetists’ experiences of postgraduate learning. The interview guide included open-ended prompts addressing clinical learning, supervision, reflection, and professional development (Supplementary Table 1). Follow-up questions were used as needed to clarify participants’ meanings and to encourage elaboration based on their responses.

Interviews lasted between 20 and 58 minutes and were conducted face-to-face by an experienced qualitative researcher. All interviews were audio-recorded and transcribed verbatim.

### Sample adequacy and variation

Twenty-nine participants were included, consistent with phenomenographic research, which seeks to capture maximum variation in ways of understanding a phenomenon rather than to achieve numerical representativeness. Adequacy was judged by the completeness and stability of the outcome space—that is, whether further data would contribute any new, qualitatively distinct conceptions [12,13]. As analysis progressed, such novel understandings became infrequent, indicating that the range of perspectives was sufficiently comprehensive. This judgement was supported by reflexive team discussions and informed by the principle of information power [14], taking into account the study’s focused aim, participant diversity, and richness of dialogue.

### Data analysis

We analysed the data using the phenomenographic method described by Dahlgren and Fallsberg [15]. The first author (HC) led the analysis and familiarised herself with all transcripts through repeated readings to develop a holistic understanding of variation in how the implicit curriculum was understood and experienced.

Following this initial familiarisation, statements reflecting different ways of understanding the implicit curriculum were identified and compared across interviews. Through iterative grouping and regrouping, similarities and differences in how the implicit curriculum was understood were discerned. Preliminary categories of description were developed and refined through constant comparison with the data, with attention to both the referential (what was understood) and structural (how attention was directed) aspects.

As analysis progressed, the categories were clarified and delimited in relation to one another, leading to the identification of three qualitatively distinct ways of understanding the implicit curriculum. These were subsequently organised into a coherent outcome space representing the collective variation in how the implicit curriculum was experienced within postgraduate anaesthesia training.

A senior phenomenographic researcher (ÅI) contributed through repeated engagement with the data and critically examined emerging interpretations, categories of description, and their structural relationships. Throughout the analytic process, all authors engaged in reflexive discussions, identifying and comparing statements that reflected different ways of understanding and engaging with the implicit curriculum and iteratively refining categories of description. Through this dialogic process, the whole research team agreed on a stable outcome space.

### Reflexivity

Interpretation was understood as a co-construction between researchers and data, consistent with the non-dualistic stance of phenomenography. The research team combined clinical and educational expertise, fostering dialogue between insider and outsider perspectives. Clinician–researchers contributed experiential insight into anaesthetic practice and its tacit norms, while educational researchers offered critical distance and theoretical grounding. Ongoing reflexive dialogue throughout the analytic process helped maintain interpretive rigour and ensure that findings remained grounded in participants’ accounts.

### Ethical considerations

The interviews analysed in this study were conducted within two phenomenographic projects on postgraduate anaesthesia training. For both original projects, the relevant ethical review boards in Sweden (reference Dnr 2020-06247) and England confirmed that formal ethical approval was not required. Both studies were conducted in accordance with the Declaration of Helsinki, and all participants provided informed consent for their data to be used in research on anaesthesia education. The present analysis remained within the scope of those approvals and consents.

## Results

### Outcome space: qualitatively different ways of conceiving the implicit curriculum

Analysis of the 29 interviews revealed three qualitatively distinct ways of understanding and relating to the implicit curriculum in postgraduate anaesthesia training. These conceptions differ in both what the implicit curriculum is understood to be (referential aspect) and how attention is directed toward it in practice (structural aspect).

The three conceptions are:

- Orientation toward the explicit curriculum
- Awareness of the implicit curriculum
- Engagement with the implicit curriculum

These conceptions do not represent stages of development or categories of individuals, but different ways in which the implicit curriculum is understood and attended to in descriptions of learning. Individual participants articulated more than one conception across different situations.

Variation across the conceptions was most clearly described across five dimensions of professional learning: communication, collaboration, decision-making, autonomy, and emotional intelligence. Together, the conceptions and dimensions constitute the study’s outcome space (Table 3).

**Table 3:** Summarises the three conceptions of the implicit curriculum and their variation across five dimensions of learning.


CONCEPTIONS →	ORIENTATION TOWARD THE EXPLICIT CURRICULUM	AWARENESS OF THE IMPLICIT CURRICULUM	ENGAGEMENT WITH THE IMPLICIT CURRICULUM

DIMENSIONS OF VARIATION ↓			

Communication	Communication learned through **formal frameworks** and feedback. *‘…break down into physical dexterity skills and leadership and communication skills’* (E2)	Communication shaped by **observation and peer interactions**. *‘You see how people de-escalate tension in a team or how they clarify misunderstandings…’* (E9)	Communication **adapted flexibly** to team, patient, and context. *‘…able to tailor that communication both to the surgeon that they’re working with, the rest of the theatre team, the patient, the trainees…’* (E5)

Collaboration	Collaboration defined by **hierarchical roles**. *‘There’s a lot of time spent one-to-one with your seniors, which is an amazing way to learn skills really thoroughly…’* (E2)	Collaboration involves **mentorship and dialogue**, but within structured roles. *‘I never feel that I’m standing there pondering and coming up with things on my own….’* (S2)	Collaboration experienced as **shared expertise and flat hierarchy**. *‘…it’s the flattening of hierarchy and saying, well as a consultant, I don’t necessarily know more.’* (E2)

Decision-making	Decisions follow **protocols and authority**. *‘I think about physiology and pharmacology and then I think about the guidelines…’* (S10)	Decisions integrate **situational awareness and team input**. *‘…most of that sort of procedural learning has a relatively baseline standard…There are other more challenging things…’* (E5)	Decisions balance **pattern recognition, flexibility, and judgement**. *‘It’s that feeling that guides all my choices…. It’s of course the sum of previous experiences’* (S13)

Autonomy	Autonomy = **structured supervision**. ‘…*there are a number of core skills that you need to establish and get under your belt…’* (E2)	Autonomy grows through **incremental responsibility**. ‘*You learn where your limits are. At first, you ask for help with everything. Then you start making small decisions on your own.’* (E8)	Autonomy = **self-directed learning and confident judgement**. *‘…if one had been completely passive, I don’t think one would have gotten there…’* (S3)

Emotional intelligence	Emotional intelligence framed by **formal feedback**. *‘Even better, afterwards we can sit down and discuss what I found difficult, and he can give me feedback’* (S10)	Emotional intelligence refined through **reflection and peer input**. *‘Okay, well, what would I do differently in that scenario again?’* (E8)	Emotional intelligence developed through **empathy, resilience, and self-awareness**. *‘Allowing your own reactions to the challenges we face to influence the treatment, actions, or how you lead the team…’* (S12)


### Conception 1: Orientation toward the explicit curriculum

#### Referential aspect

In this conception, learning is understood primarily as engagement with the formal and visible curriculum. Competence is equated with acquiring predefined skills, meeting assessment criteria, and progressing through structured supervision. Learning is understood as something that can be taught, assessed, and confirmed through external standards.

This conception is reflected in accounts where learning is experienced as externally verified achievement:

*“I like taking exams. I enjoy that visual confirmation of what I know — getting it in black and white that I actually know something. That’s reassuring.”* (S2)

Here, learning is experienced as confirmation through assessment, where what counts as knowing is that which can be made visible and validated, and where educational significance is located in assessment outcomes. A similar meaning is evident in descriptions of competence as the accumulation of formally recognised skills:

*“There are a number of core skills that you need to establish and get under your belt before you can really move on.”* (E2)

In this way, learning is understood as sequential acquisition, where progress is defined in relation to explicit requirements rather than to contextual judgement.

#### Structural aspect

Attention is directed toward external markers of competence, such as guidelines, protocols, assessment frameworks, and supervisors’ instructions. Learning is experienced as alignment with explicit expectations through instruction, feedback, and verification, rather than through interpretation of social, relational, or emotional cues. These aspects of practice are foregrounded, while tacit norms, relational dynamics, and emotional aspects of practice remain backgrounded in descriptions of learning.

This orientation becomes particularly visible in situations where senior clinicians alter a planned approach without explanation:

*“You plan to give anaesthesia in a certain way and then the specialist comes and says, ‘No, I want you to do it like this,’ without any real reason — it’s just how they usually do it. The worst is when nothing is said until you’re in theatre, when the patient is already there.”* (S7)

In this account, the unspoken rationale guiding the specialist’s decision is not taken up as learning; responsibility is attributed externally, and the implicit judgement remains backgrounded. The situation is experienced as a disruption of expected explicit guidance rather than as an opportunity for learning, reflecting an understanding of learning that privileges explicit explanation over tacit modelling.

### Conception 2: Awareness of the implicit curriculum

#### Referential aspect

In this conception, participants describe learning as extending beyond an exclusive focus on the formal curriculum, to include awareness of implicit expectations and norms embedded within both formal and informal aspects of practice. The implicit curriculum becomes recognisable through observation, participation in team interactions, and exposure to clinical complexity. Formal knowledge remains important but is insufficient alone; learning requires interpretation and contextual judgement.

This conception is reflected in accounts where learning is understood as encompassing aspects of practice that are not formally specified but are nevertheless integral to professional work:

*“…there are other skills that are not directly taught or assessed but that you kind of learn along the way. Communicating properly with the patient, managing your theatre team, ensuring that you and your ODP are on the same wavelength… those are skills as well, and those are just learned along the way.” (*E13)

Here, learning includes relational and interactional aspects of practice that are not explicitly articulated within the formal curriculum but are recognised as part of what counts as learning in clinical work.

#### Structural aspect

Attention alternates between explicit guidance and implicit cues. Participants attend not only to protocols and recommendations, but also to patient-specific factors, situational constraints, and how clinicians adapt formal guidance in practice. Learning is experienced as layered and context dependent. However, in this conception, recognition of implicit expectations is foregrounded over responsibility for action. Participants describe noticing unspoken expectations and limits while responsibility for intervening or shaping outcomes remains in the background.

This orientation becomes particularly visible in accounts where participants describe noticing implicit expectations governing communication and emotional conduct:

*“You see how people de-escalate tension in a team, how they clarify misunderstandings without making it worse. No one tells you ‘this is how you do it’, but you notice that there’s an expectation that you keep things calm, especially in theatre. You just pick that up by watching how things unfold.”* (E9)

Here, learning involves recognising a tacit normative expectation regarding emotional regulation and relational restraint. The participant notices how certain ways of acting are implicitly valued but does not describe assuming responsibility for directing or shaping team dynamics.

### Conception 3: Engagement with the implicit curriculum

#### Referential aspect

In this conception, the implicit curriculum is understood as integral to professional practice, shaping judgement and action. Learning is understood as active engagement with tacit norms, emotional responses, and moral expectations, which are integrated with formal knowledge to guide action in complex situations. Learning is characterised by a sense of responsibility for responding to implicit concerns, rather than merely recognising them.

This conception is, for example, reflected in accounts where learning involves taking up responsibility within practice through participation in tacitly structured roles:

*“When I’m on Neuro, I’m usually the one responsible for the theatres. The consultant steps back so that I take the questions and manage the communication with other doctors and nurses. That’s how trust is built — and how I end up making more decisions.”* (S11)

Here, learning is understood as taking up responsibility in practice in relation to tacit expectations of authority and trust, reflecting an understanding of learning as inseparable from professional agency.

#### Structural aspect

Attention is directed toward tacit and relational dimensions of practice, which move into the foreground. Formal guidelines remain present but are backgrounded, functioning as resources rather than determinants of action. The relationship between practitioner and context is experienced as reciprocal, with practice understood as something that can be shaped through leadership, communication, and intervention when implicit concerns require explicit action.

This orientation becomes particularly visible in accounts where participants describe acting on tacit concerns in the absence of explicit instruction:

*“If I hadn’t spoken about my concerns, we would have stayed in that situation where everyone had tunnel vision. Just vocalising those concerns as a team lets us actually do the right thing for the patient.”* (E8)

Here, attention is directed toward a tacitly recognised concern that is taken up as grounds for action, with responsibility for intervening foregrounded as part of what is recognised as learning.

### Summary of variation across conceptions

Across the three conceptions, anaesthetists described qualitatively different ways of understanding and relating to the implicit curriculum: from reliance on explicit structures, through recognition of tacit expectations, to active engagement involving responsibility for interpretation and action. In broad terms, engagement with the implicit curriculum was more often described in relation to greater experience and responsibility. However, these patterns did not reflect a developmental sequence: participants across training levels expressed multiple conceptions depending on the situation.

The five dimensions—communication, collaboration, decision-making, autonomy, and emotional intelligence—illustrate how these ways of understanding are expressed in practice.

Across all three conceptions, participants described situations in which formal curricular structures did not fully capture what was implicitly expected. What differed was how such misalignments were interpreted and acted upon: as confusing deviations from formal rules, as cues requiring interpretation, or as grounds for intentional negotiation or departure based on contextual judgement.

Additional illustrative quotations for each dimension are provided in Supplementary Table 2.

## Discussion

### Principal findings

This study explored how anaesthetists experience the implicit curriculum in postgraduate education. Using a phenomenographic approach, we identified three qualitatively distinct ways of conceiving the implicit curriculum: orientation toward the explicit curriculum, awareness of the implicit curriculum, and engagement with the implicit curriculum.

A central finding of this study is that explicit and implicit curricular dimensions are experienced as intertwined in everyday clinical work, rather than as separate layers of training. What varied was not the presence of implicit influences, but which aspects of practice were recognised as curricular and taken up as learning. In some experiences, curriculum was understood primarily in terms of what was specified and assessed; in others, learning was constituted through relational, situational, and often uncertain aspects of practice—such that the implicit curriculum effectively functioned as *the* curriculum.

From this perspective, the implicit curriculum did not appear as a hidden layer beneath formal training, but as part of how learning was enacted in practice. Differences arose not from unequal access to learning opportunities, but from differing assumptions about what counted as curriculum and how it was encountered. In relation to current emphases on transparency and standardisation, these findings suggest that formalisation alone does not ensure shared recognition of what is educationally meaningful in clinical work.

### What this study adds to existing literature

Empirical research on the hidden and informal curricula, and related scholarship on tacit and implicit influences in clinical learning, has shown that professional learning in medicine is shaped not only through formal teaching, but through the tacit transmission of norms, values, expectations, and judgements embedded in everyday clinical practice [7,9,16]. Studies across medical education have examined how such implicit influences are enacted in practice—through communication, teamwork, role modelling, and feedback—shaping professional identity, judgement, and participation in clinical work [17,18,19]. Similar processes have been demonstrated in qualitative studies within anaesthesia, where expertise and non-technical skills develop through tacit processes such as observation, situational judgement, and progressive participation in everyday clinical work [20,21,22,23]. Together, this body of work has established the importance of the implicit curriculum while recent reviews have highlighted challenges in conceptualising and investigating it empirically [24].

Much of the literature on the hidden curriculum has conceptualised tacit curricular influences as embedded within learning environments and institutional cultures. Within this framing, scholarship has often foregrounded problematic influences, with comparatively less attention to implicit influences that may be supportive or reinforcing of professional learning [25]. While this framing has generated rich descriptions of how implicit influences shape professional practice, it has offered less insight into how such influences are recognised and taken up as curriculum by learners themselves, or why the same clinical practices may be experienced as educationally meaningful in different ways.

The present study addresses this gap by examining variation in how anaesthetists experience the implicit curriculum in postgraduate training. The findings show that communication, collaboration, decision-making, autonomy, and emotional intelligence are not uniformly experienced as learning; rather, their educational significance varies according to whether these aspects of practice are approached as formally specified skills, as tacitly interpreted through participation, or as flexibly enacted through judgement and agency in context. In this way, the implicit curriculum is not treated as a fixed feature of the learning environment, but as something that comes to be recognised as curriculum through experience.

This perspective complements sociocultural accounts of workplace learning that emphasise participation, interaction, and situated activity as central to professional development [26,27]. It suggests that participation alone does not account for how implicit curricular influences shape learning, and that professional capability is shaped through qualitative differences in what learners attend to and take up as curriculum in everyday clinical work.

### Practical and educational implications

The findings of this study do not suggest that the implicit curriculum should be made explicit, formalised, or directly assessed. Rather, they highlight how differences in what is recognised as curriculum shape how learning is understood and enacted in practice. The implications therefore concern how educators interpret, frame, and engage with everyday clinical work as educational, rather than how they intervene in it.

#### Supervision as alignment of educational meaning

From this perspective, supervision can be understood as a process through which the educational meaning of clinical actions is aligned between trainees and supervisors. Clinical activities do not inherently signify learning; what they represent depends on assumptions about what counts as curriculum in a given situation.

For example, a decision to deviate from a guideline may be experienced as either a failure to follow protocol or as an exercise of professional judgement. These reflect different interpretations of their educational significance. Supervision, therefore, involves making space to surface and negotiate these interpretations, rather than prescribing a single correct meaning.

#### Reflection as expanding what is recognised as learning

Reflection is often positioned as a way to consolidate learning from experience. The present findings suggest an additional role: reflection as a means of expanding what is recognised as legitimate learning in clinical work. Across the outcome space, differences in learning were not driven by access to experiences, but by whether relational, emotional, and situational aspects of practice were recognised as curricular. Reflective dialogue can support learning by helping trainees articulate what they attend to as learning and gradually extending that recognition to include tacit and value-laden aspects of practice—without formalising them.

#### Assessment as interpretation rather than capture

The findings also suggest caution in how assessment practices are interpreted. Similar observable behaviours may reflect qualitatively different understandings of practice, depending on how trainees conceptualise decision-making, collaboration, or autonomy. Broadening assessment, in this sense, does not require measuring the implicit curriculum, but attending to how trainees explain, justify, and account for their actions across different interpretations of practice.

This approach foregrounds judgement, meaning making, and responsibility, while resisting the impulse to translate tacit learning into new formal criteria.

### Strengths and limitations

A key strength of this study lies in its phenomenographic design, which captures qualitative variation rather than assuming linear progression. It shifts attention from describing the implicit curriculum to understanding how it becomes educational through learners’ experience and engagement.

The analysis drew on interview material not originally focused explicitly on the implicit curriculum, which may have constrained the depth with which some aspects were articulated, although variation in ways of understanding was still discernible across the collective dataset. Phenomenography maps variation rather than prevalence; hence the findings show how anaesthetists understand the implicit curriculum, not how many share each view. Conducted within anaesthesia, the study reflects a specialty with particular relational and cognitive demands, but its conceptual insights are likely to resonate across postgraduate medicine; implications for other contexts should therefore be interpreted by analogy rather than direct transfer.

These limitations also suggest future directions: the implicit curriculum is best examined through complementary, longitudinal, and cross-contextual approaches that capture its evolving and situated nature.

### Future research

Further work could explore how awareness of the implicit curriculum develops over time and what educational or organisational factors support its growth. Longitudinal or cross-contextual designs could capture how engagement with tacit learning evolves with experience, cultural context, or institutional change.

Future research might also investigate faculty development initiatives that help supervisors articulate and discuss implicit learning without over-formalising it. Comparative studies across specialties could identify which tacit influences are context-specific and which are common to postgraduate medicine as a whole.

Finally, as training environments become increasingly mediated by digital technologies and AI-driven assessment, researchers should examine how automation interacts with tacit dimensions of learning—and how professional formation can remain human, relational, and interpretive within these emerging systems.

## Conclusion

This study shows that the implicit curriculum is a lived dimension of postgraduate anaesthesia training, encountered in the daily interactions and unspoken expectations that define professional life. By identifying three distinct ways anaesthetists relate to these influences, the study provides an empirically grounded outcome space that captures variation in awareness and engagement.

This framework can guide educators in recognising and supporting diverse approaches to learning, helping make the implicit curriculum a visible, discussable, and intentional part of postgraduate education. As training becomes increasingly structured and technologically mediated, fostering awareness of tacit learning processes will be vital to preserving the reflective and relational dimensions of clinical expertise, without formalising what is inherently tacit.

## Artificial Intelligence use

During the preparation of this manuscript, the authors used the AI-based language model ChatGPT (OpenAI) to assist with language refinement, clarity, and consistency of phrasing. The authors reviewed and edited all suggestions to ensure accuracy and integrity and take full responsibility for the content of the manuscript.

## Additional Files

The additional files for this article can be found as follows:

10.5334/pme.2224.s1 Supplementary Table 1. Example open interview questions.

10.5334/pme.2224.s2 Supplementary Table 2. Illustrative quotations for the “Engagement with the implicit curriculum” conception across five dimensions of learning.

## Data Availability

The interview data are not publicly available due to participant confidentiality.
