# Supplementary material for: Learning Between the Lines: Anaesthetists’ Conceptions of the Implicit Curriculum in Postgraduate Education
Source: Perspect Med Educ. 2026 Jun 15;15(1):512–22. doi: 10.5334/pme.2224 (PMC13281737; doi:10.5334/pme.2224)
Supplement: Supplementary Table 1. — Example open interview questions. [file pme-15-1-2224-s1.pdf]

Supplementary Table 1. Example open interview questions

Open interview questions
Describe the specialty training programme to a doctor new to anaesthesia.<br><br>How does this postgraduate programme differ from others you have experienced?<br><br>If you could design your own training programme, what would it look like?<br><br>Describe a day that was highly educational, and one that was not.<br><br>Describe an excellent anaesthetist—and a poor one.

Table listing example open interview questions used in the study.
