# Supplementary material for: Learning Between the Lines: Anaesthetists’ Conceptions of the Implicit Curriculum in Postgraduate Education
Source: Perspect Med Educ. 2026 Jun 15;15(1):512–22. doi: 10.5334/pme.2224 (PMC13281737; doi:10.5334/pme.2224)
Supplement: Supplementary Table 2. — Illustrative quotations for the “Engagement with the implicit curriculum” conception across five dimensions of learning. [file pme-15-1-2224-s2.pdf]

Supplementary Table 2. Illustrative quotations for the "Engagement with the implicit curriculum" conception across five dimensions of learning

This table provides verbatim quotations illustrating how anaesthesia trainees experienced and understood *Engagement with the implicit curriculum*, the most inclusive conception identified through phenomenographic analysis. Each dimension is described in terms of (a) contextual aspects of experience and (b) reflective processes of meaning-making.

Dimensions of variation	Contextual Aspects of Experience	Reflective Processes of Meaning-Making	Illustrative Quotations
**Communication**	Learning communication through everyday team interactions, informal mentoring, and unspoken workplace norms; adapting communication fluidly to hierarchy, patient needs, and context.	Reflection on tone, emotion, and interpersonal awareness; recognising communication as part of relational practice and professional judgement.	*'In a tense situation, the excellent anaesthetist can raise their voice without anyone feeling overwhelmed; instead, everyone finds it clear and effective. In a calm situation, it's the kind of colleague who can delegate tasks to the completely new ones and do so in a way that makes them feel safe.'* (S13)<br><br>*'It is kind of exemplary and they're able to tailor that communication both to the surgeon that they're working with, the rest of the theatre team, the patient, the trainees, the health care assistant that comes in, so deals with everybody in a slightly bespoke way but kind of adds equal value to everybody that comes in.'*(E5)
**Collaboration**	Teamwork experienced as shared expertise and psychological safety; contribution valued across roles and experience levels.	Recognition of mutual learning; seeing oneself as an active participant rather than a learner positioned below others.	*'Maybe, it could be that you've learned something about yourself, or how you act in a group and so on. I find that much harder, you know, by myself. Yes, sometimes you also value whether you really learned something, if you became better when you were here, and if you've become better at collaborating with the group.'* (S15)<br><br>*'At first, I second-guessed every instruction I gave. But after a while, you start trusting your instincts and your colleagues.'* (E9)
**Decision-making**	Judgement experienced as integrating formal knowledge with situational awareness and dialogue; flexibility within, not against, institutional expectations.	Developing intuitive and context-sensitive reasoning through reflection on complexity and uncertainty.	*'Yeah, so I think, in ICU especially, we look for patterns. We look for patterns of clinical cause. We look for patterns of lab results, patterns of, you know, various diagnostics and also time frame, so chronology, of how patients … And I think it's that pattern of progression of clinical cause, the trajectory and the speed of the progression, that …'* (E12)<br><br>*'It's also difficult with what we know in this field,*

			that you think you know something, and then it no longer applies after a while. I think it's both quite enjoyable, because then you get to learn something new, but it can also be quite frustrating when you think, "But we had a great model for this," and then it doesn't work.'(S15)
**Autonomy**	Autonomy experienced as graduated responsibility within supportive supervision; independence grows through trust and shared accountability.	Reflection on confidence and capability; understanding autonomy as negotiated rather than granted.	*'He (senior consultant) does something else because he wants me to handle these questions and manage communication with other doctors, nurses, emergency list, etc. In this way, it increases the trust in me, and I get to do more and maybe even have more decision-making power.'* (S11)<br><br>*'It's something that somehow comes to you, and when you master it, it's just there. And I believe it's the same with our things as well, even though it's more theoretical. The more you do it, and it's not just enough to do something, but you should really want to.'* (S15)
**Emotional intelligence**	Emotional learning experienced through informal feedback, observation, and modelling of empathy and composure by colleagues.	Developing awareness of emotional impact on judgement and relationships; balancing empathy with professional distance.	*'I think you need a huge amount of empathy and kindness around, you know, looking after people who are unwell, so guiding them through their illness, being able to convey relevant information and provide support to family and friends and also to other clinical staff. We're called to see people in situations that many other specialties would find very scary, is the reality, I suppose.'* (E5)<br><br>*'You will be a worse doctor if you constantly start relating to your own child instead of the child you are caring for. Then you will be much, much worse.'* (S13)
